# Medication-related osteonecrosis of the jaw associated with implant and regenerative treatments: Systematic review

**DOI:** 10.4317/medoral.22691

**Published:** 2019-03

**Authors:** António Granate-Marques, Carlos Polis-Yanes, Maria Seminario-Amez, Enric Jané-Salas, Jose López-López

**Affiliations:** 1 DDS, Master’s degree, School of Dentistry, University of Barcelona. University Campus of Bellvitge, Barcelona, Spain; 2 DDS, Professor of Master’s degree, School of Dentistry, University of Barcelona. University Campus of Bellvitge, Barcelona, Spain; 3 DDS, MD, PhD, Department of Odontoestomatology. Faculty of Medicine and Health Sciences (School of Dentistry), University of Barcelona. University Campus of Bellvitge, Barcelona, Spain. / Dental Hospital University of Barcelona, (Barcelona University) / Oral Health and Masticatory System Group (Bellvitge Biomedical Research Institute) IDIBELL, Barcelona, Spain

## Abstract

**Background:**

The aim of this study was to determine if the treatment with bisphosphonates other anti-resorptive and antiangiogenic agents influences the success of regenerative and / or implant treatments.

**Material and Methods:**

We reviewed the literature from the last 5 years in the PubMed database, using the following words: “Sinus Floor Augmentation”[Mesh] OR “Dental Implants”[Mesh]) OR “Guided Tissue Regeneration”[Mesh]) AND “Osteonecrosis”[Mesh]. The articles were selected following the inclusion and exclusion criteria and were evaluated using the 22 items of the STROBE declaration. The following PICO clinical question was applied: Does the treatment with agents associated with drug osteonecrosis influence the success of regenerative and implant treatments?

**Results:**

The initial search resulted in a total of 27 articles. After eliminating those that did not refer to the topic, were duplicated or did not meet the inclusion / exclusion criteria, a full reading of the articles was made evaluating their methodological quality, obtaining six studies with high methodological quality and two with moderate.

**Conclusions:**

The literature regarding this topic is scarce, randomized clinical trials would be necessary to establish protocols relative to implant treatment in patients on antiresorptive treatments. The risk of developing an osteonecrosis associated with the regeneration / implant placement in patients with benign bone diseases is scarce, but it exists and it should not be underestimated. Especially, in the posterior areas of the jaw, if the duration of treatment with BP is greater than 3 years, and if the patient is under therapy with systemic corticosteroids.

** Key words:**Bisphosphonates, monoclonal antibodies, implants, sinus lift, guided bone regeneration, osteonecrosis.

## Introduction

The term “Osteonecrosis of the jaws” was introduced by Marx in 2003 (1) and later by Ruggiero *et al.* in 2004 (2,3). This term refers to bone exposures (in the maxilla or mandible) without early healing, associated with the use of bisphosphonates (BP). Bagán *et al.* describes the same lesion associated with antiresorptive medication in his series of 10 clinical cases, using the term avascular bone necrosis (4).

To be considered a Bisphosphonate-related osteonecrosis of the jaw it should persist for at least 6-8 weeks in the absence of radiation therapy in the affected area; and it may – or may not - be associated with high morbidity, pain, tooth mobility, halitosis, paresthesia, bone sequestration and intraoral or extraoral fistula (5,6).

Bisphosphonates are frequently used to modulate the bone remodeling cycle in benign bone disorders such as osteoporosis, osteogenesis imperfecta and Paget’s disease. They are also used to prevent and control the bone activity of certain malignant neoplasms, such as multiple myeloma and bone metastases of prostate cancer, breast cancer, among others (7). According to their action mechanism, they are divided into two main groups: the first generation (non-nitrogen): etidronate, clodronate and tiludronate; and those of second and third generation (with-nitrogen): alendronate, risedronate, ibandronate, pamidronate and zoledronic acid. The route of administration includes the oral and parenteral route (7,8).

The oral BPs have a low absorption rate and have a short half-life (between 30 and 120 minutes), with 20 to 80% of the substance deposited in the bone (9). However, those of intravenous use have a high bioavailability and once absorbed in the bone tissue, it can take more than 10 years to metabolize, and consequently excrete. The BP that have a higher binding affinity, in descending order, are: zoledronic acid > alendronate > ibandronate > risedronate > etidronate (8,10). According to the Advisory Task Force Group on Bisphosphonate-related Osteonecrosis of Jaws until 2007, 190 million treatments with bisphosphonates were prescribed worldwide (11). These drugs have different common adverse effects: esophageal ulcer, atypical fracture of the femur, atrial fibrillation and osteonecrosis of the jaw (5,12). Although BP are the main pharmacological cause of osteochimionecrosis; there are other drugs frequently used, especially in recent years, in the treatment of osteoporosis and bone metastasis; called Anti RANKL monoclonal antibodies that can cause the same adverse effect. One of these agents is Denosumab (Prolia®, Xgeva®) a highly specific human IgG2 monoclonal antibody to the receptor activator of nuclear factor-B ligand (RANKL), inhibiting the activity of osteoclasts, reducing resorption and increasing bone density. Bevacizumab (Avastin®) and Sunitinib (Sutent®), two antiangiogenic drugs, are frequently used in oncological patients and can be involved in the pathogenesis of Medication-related osteonecrosis of the jaw (MRONJ) (13-16).

Ruggiero et al. in an update article by the American Association of Oral and Maxillofacial Surgeons (AAOMS) mentions that the incidence of osteonecrosis is higher in patients who received high doses of BP during the treatment of neoplastic diseases with metastasis with an incidence of 1 to 10%. This is most likely associated with the frequency and dose of the BP prescribed (17-19). On the other hand, in the few studies available, it has been confirmed that the risk of Osteonecrosis in patients receiving BP for the treatment of osteoporosis is very low, between 1 / 10,000 and 1 / 100,000 patients / treatment and year (13,17,20). Patients receiving BP or Denosumab therapy for the treatment of bone metastases and multiple myeloma receive monthly doses of intravenous (for BP) or subcutaneous (for Denosumab). While patients with osteoporosis or other bone disease such as Paget’s disease (osteitis deformans) require antiresorptive therapy at much lower doses (Denosumab 60mg / 6months, zoledronic acid 5mg every 1 to 5 years) (21).

Ruggiero & Drew (22) propose a classification that allows the categorization of an Osteonecrosis into different stages according to the severity of its signs and symptoms. Subsequently, the AAOMS adapts this classification including a stage 0 and the patient at risk (3) ( Table 1). Alternatively, Bagán et al. considered two subdivisions within stage II (23). The diagnosis of Osteonecrosis is based mainly on the presence of the following characteristics: patient under treatment with BP, areas of bone exposed without healing more than 6-8 weeks and absence of radiotherapy in the craniofacial region (19,24).

**Table 1:**
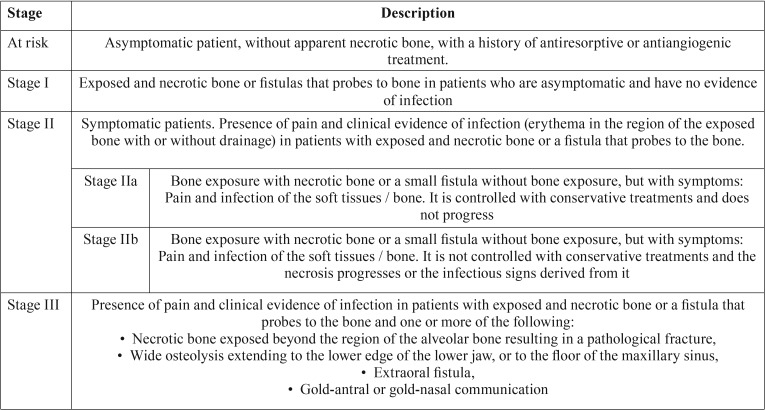
Staging of Osteochimionecrosis according to the AAOMS and subdivision proposed by Bagán *et al.* (23).

Risk factors can be divided into two groups, local factors, which include surgical treatments (eg, dental extractions and surgical periodontal procedures) and concomitant oral diseases (eg, periodontal diseases, tooth decay and abscesses), and systemic factors, such as advanced age, tobacco use, corticosteroid therapy, and coexisting conditions such as anemia and diabetes (25-27). According to the AAOMS, the risk of developing osteonecrosis in patients who have been exposed to antiresorptive medications for other dentoalveolar operations, such as dental implant placement and endodontic or periodontal procedures is unknown (19). According to the literature implant surgery should be avoided in patients under intravenous treatment with BP. For patients undergoing oral BP treatment for less than 3 years dental implants can be inserted safely (19). However, the patient should always be warned of possible immediate or late risk (19). Most authors agree that the risk is closely related to the patient’s condition and the duration of treatment with antiresorptive agents, but there is an increasing number of clinical cases of Osteonecrosis after the placement of dental implants (24). Given the current controversy on the subject, our main objective is to revise the published studies assessing the prognosis of rehabilitation treatments with dental implants (with or without previous bone regeneration) in patients pre or post treatment with antiresorptive agents. The results observed were the implant loss, the failure of the regenerative treatments and the incidence of Osteonecrosis.

## Material and Methods

A bibliographic review was made in the PubMed database during the last 5 years using the following words: “Sinus Floor Augmentation”[Mesh] OR “Dental Implants”[Mesh]) OR “Guided Tissue Regeneration”[Mesh]) AND “Osteonecrosis”[Mesh]. The articles included were retrospective, prospective observational studies and series of clinical cases focused on the subject, including patients undergoing treatment with agents associated with drug osteonecrosis and submitted to a bone guided regeneration procedure and / or dental implants, written in English, Spanish or Portuguese, evaluated on humans. Experimental laboratory studies; animal studies; studies in which the main topic was not the relationship between dental implants and / or regeneration and systemic therapy with antiresorptive agents and duplicate articles, were excluded from the review. Two authors (AMG and CPY) independently reviewed all articles and extracted data from each study including first author, publication year, population sampled, type of study, gender, age / average range, risk factors, follow-up, indication of the use of bisphosphonate, administration route, type of bisphosphonate, duration of treatment, incidence of MRONJ, stage of MRONJ, location of MRONJ, lost implants, position of the lost implants and type of graft. The results obtained by AMG and CPY were compared and reviewed by a third researcher (MAS). The authors (EJS y JLL) reviewed the final draft of the manuscript as a further quality check.

The eight articles reviewed were evaluated using the 22 items of the STROBE declaration (STrengthening the Reporting of OBservational studies in Epidemiology). The studies that presented at least 15 of the 22 criteria were considered as having a “high methodological quality “; those who presented 8 to 14 of the criteria were considered as having “moderate methodological quality”; and those studies that presented less than 7 criteria were considered as “low methodological quality” (28).

This article was written according to the PRISMA statement (Preferred Reporting Items for Systematic Reviews and Meta-Analyzes for Protocols) and the following PICO clinical question was used: Does the treatment with agents associated with drug osteonecrosis influence the success of regenerative and implant treatments? (29).

## Results

**Figure 1:**
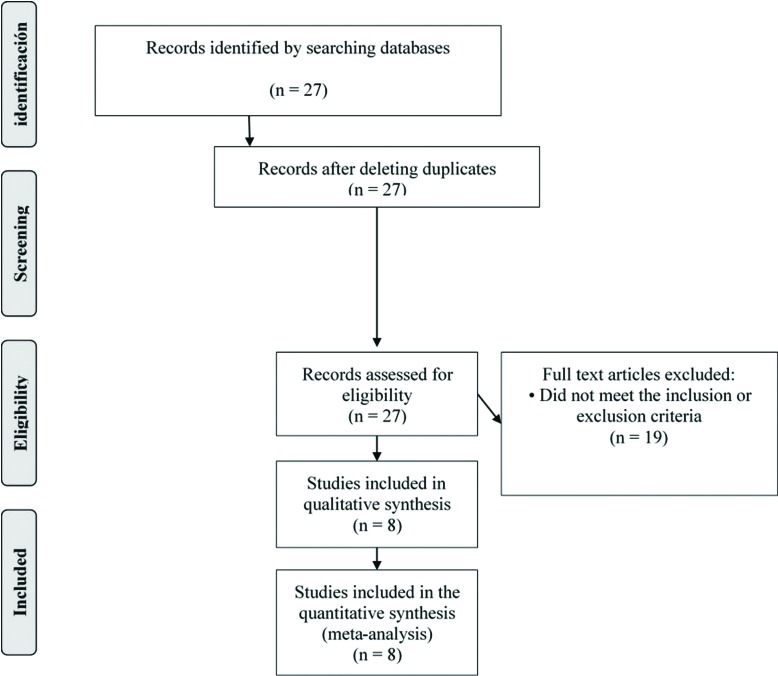
Diagram of the search process and results.

In the initial search we obtained a total of 27 articles with which a manual review was made by reading the summaries, in order to exclude all those that did not refer to the topic, were duplicated or did not meet the inclusion / exclusion criteria, from this review we eliminated 19 articles (Fig. 1). A full reading of the articles was made, evaluating their methodological quality, obtaining a total of 8 articles, 6 of them with a high methodological quality and 2 with moderate ( Table 1). We included in this review all articles of high and moderate quality, obtaining 6 observational, descriptive and retrospective series of clinical cases; 1 Observational, Descriptive and prospective case series and 1 Cohort Prospective Analytical Study. 22 articles were included due to their relevance and interest for the subject, 11 are reviews of the literature, 5 are systematic reviews, 2 retrospective cohort studies, 2 meta-analyzes, a series of clinical cases and a letter to the editor.

In  Table 2 we present a summary of the data that we consider most relevant in the studies reviewed.

**Table 2:**
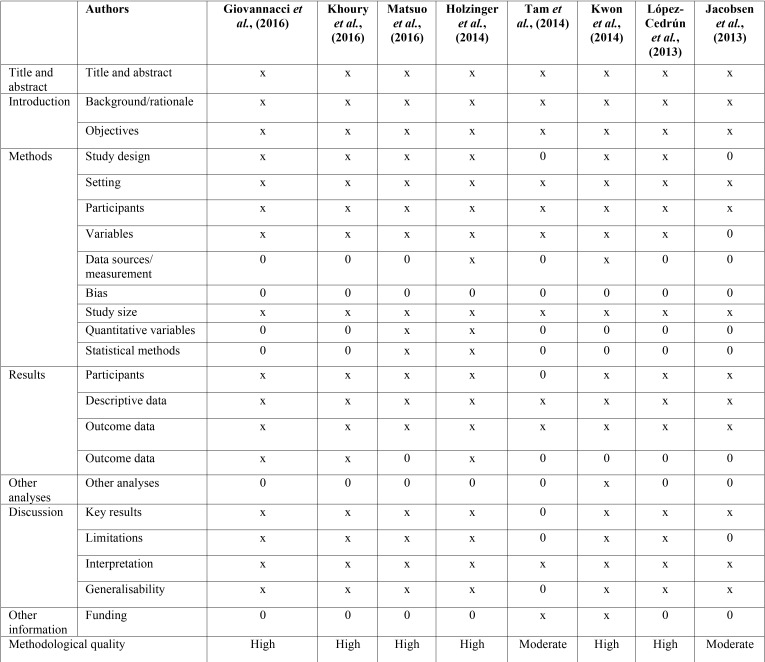
Evaluation following the criteria of the STROBE Declaration (x - Meets the criteria; 0 - Does not meet the criteria).

From the collected data we obtained a sample of 135 patients, the great majority being women (n = 81) in which the age varies between 42 and 79 years. The main indication and method of administration for the use of bisphosphonates was osteoporosis and the oral route, except in the study by Matsuo *et al.* that used a sample of oncological patients in treatment with intravenous bisphosphonates. Eighty-two patients developed osteonecrosis associated to implants, with a higher prevalence in the posterior sectors of the maxilla (n = 22) and mandible (n = 33). Forty patients were under treatment with IV BP (Average of Duration of BP treatment: 44 months) and 42 where under oral BP (Average of Duration of BP treatment: 56 months). Only one of the reviewed articles focuses on regenerative procedures.

## Discussion

In the studies included most patients under treatment with intravenous bisphosphonates were receiving zoledronic acid and the majority of patients under treatment for osteoporosis with oral bisphosphonates were taking alendronic acid (9,24-27,30). Kwon et al. and López-Cedrún *et al.* show that the majority of patients who developed Osteonecrosis associated with the placement of dental implants received alendronate orally (24,27). Holzinger *et al.* studied the temporal relationship between the occurrence of Osteonecrosis and the moment of implant placement, concluding that the risk of developing Osteonecrosis is greater and more accelerated when the implantation has been performed after the start or during the therapy with bisphosphonates (9). Within the same subject, Giovannacci *et al.* reported that the average duration of treatment with bisphosphonates before the onset of osteonecrosis was longer for patients undergoing oral bisphosphonates than patients undergoing intravenous therapy (26). Jacobsen *et al.* also reported in their study that the average duration of treatment with bisphosphonates was 38 months in patients who received intravenous BP and approximately 50 months in patients who received oral BP treatment for osteoporosis (30). On the other hand, López-Cedrún *et al.* and Giovannacci *et al.* present a longer average interval of approximately 5 and 6 years respectively (24,26).

The study by Matsuo *et al.* is particularly interesting, being the only study that focuses exclusively on cancer patients and treatment with intravenous bisphosphonates. In addition, it only assesses patients who started bisphosphonate therapy after implant treatment. In their conclusions, the risk for osteonecrosis induced by intravenous therapy with bisphosphonates should be evaluated separately in patients receiving monthly doses and those who receive an injection every 6 months, since both the primary disease and the cumulative dose are completely different between the two uses (16).

Some authors affirm that not only implant surgery, but also the existence of the implant itself, seems to be associated with Osteonecrosis (9,16,24-27). Most articles classify Osteonecrosis as “Implant surgery triggered” when Osteonecrosis occurs immediately after implant placement (2 to 10 months) and “implant presence triggered” in which Osteonecrosis developed after one year of implant placement (26). Giovannacci *et al.* in its retrospective study with 15 patients with osteonecrosis around dental implants, relates 9 of the cases to the presence of the implant itself, considering that the osteonecrosis occurred after a time interval of 1 to 15 years after the surgical procedure and subsequent successful osseointegration (26). Know *et al.* analyzed a series of 19 Osteonecrosis associated with implants in patients under therapy with BP. Of these 19, 3 patients (15.8%) developed Osteonecrosis within 6 months after implant surgery. In 5 patients (26.3%) Osteonecrosis was related to the surgical trauma of implant explantation and debridement of the bone. However, in the majority of patients (n = 11, 58%) Osteonecrosis appeared without any relation to the surgical trauma of the insertion or extraction of the dental implant (27). López-Cedrùn *et al.* in their study present a series of 9 patients medicated with oral bisphosphonates and with Osteonecrosis around the implants in which Osteonecrosis was an early complication (from 1 to 12 months) in 4 patients (44.4%) and a late complication (from 18 to 96 months) in the remaining 5 (55.6%) (24). In 4 of the 27 patients assessed by Jacobsen et al. implant insertion was performed several months before the start of BP therapy; concluding, therefore, that, in these patients, the implant itself, and not the surgical insertion, was the local factor for the development of Osteonecrosis (30).

Osteonecrosis associated with dental implants seems to preferentially affect the posterior sectors of the mandible and the maxilla and, in general, it is a late complication unrelated to the operation (24,26,30). In the study by Jacobsen *et al.* 9 of the 12 patients showed implant failure in the mandible or the posterior maxilla (30). The etiopathological process of Osteonecrosis remains unclear. However, Jacobsen *et al.* analyzed the necrotic bone of 12 patients, obtaining as an Histological result Actinomyces plates in 7 of the samples, allowing to correlate the process of Osteonecrosis with infection, also after starting the systemic antibiotic treatment, discomfort and other symptoms such as hypoesthesia resolved in all patients (30).

Kwon *et al.* defend in their article the theory that the increased risk of developing Osteonecrosis in implants already integrated in patients taking BP could be explained by the reduced response to bone remodeling considering that the bone tissue around a loaded osseointegrated dental implants is subject to continuous remodeling (27). Tam *et al.* present two cases of osteonecrosis associated with implants in patients under treatment with oral bisphosphonates and propose another justification in which the surgical trauma during implant surgery could stimulate the postoperative accumulation of the drug at implant sites in the patients who maintain bisphosphonate therapy after the surgery (25).

Diseases such as diabetes, corticosteroid treatment and smoking habit are identified as predisposing conditions for the development of osteonecrosis. However, there is no homogeneity of data in the literature. In a study by Giovannacci *et al.* the number of patients receiving corticosteroids was higher, especially among oncological patients (26). Matsuo *et al.* in a previous study using the same cohort sample of the reviewed article showed that the correlations between osteonecrosis and systemic factors were significantly low. However, certain local factors, particularly oral hygiene and oral infectious diseases (e.j. periimplatitis), showed significantly high correlations (16).

One of the groups that studied this issue, Kwon *et al.* (2013), identify three characteristic patterns of bone destruction of the Osteonecrosis lesion around the implant: i) “frozen type” - abundant bone necrosis around the implant and adjacent alveolar bone (areas of necrosis are more evident than inflammatory components of soft tissue). ii) “osteolytic” - extensive osteolysis around an implant with / without sequestration formation (increase in soft inflammatory tissue and residual necrotic or viable bone particles similar to conventional osteomyelitis). iii) “en block type” - block sequestration with the implant (a considerable amount of implant-bone contact is maintained). Six of the assessed patients presented “en block type” sequestration, in which maintenance of the osseointegration of the implant surface to the surrounding necrotic bone was observed. These findings are different from the typical bone destruction induced by peri-implant disease. “En bloc type” sequestration seems to be one of the characteristics of implant-related Osteonecrosis (27). Within the same line of investigation Tam *et al.* report in their study that most of the implants lost due to thermal damage and bacterial infection present a marginal bone loss or around the dental implant and that, in their series of cases, five patients showed bone destruction around the dental implant resembling an osteomyelitis bone sequestration (25). It is noteworthy that studies such as that of Jacobsen *et al.*, identified bacteria, especially Actinomyces, in biopsy samples of necrotic bone from patients with Osteonecrosis, this associated with the late failure of the implants leads to certain authors, including Holzinger *et al.*, to consider periimplantitis as a possible risk factor for Osteonecrosis. However, they concluded that randomized and prospective clinical trials are necessary to establish this relationship (9,16,30,31). All the suggested theories regarding MRONJ, proposed by the different authors, are summarized in  Table 3.

**Table 3:**
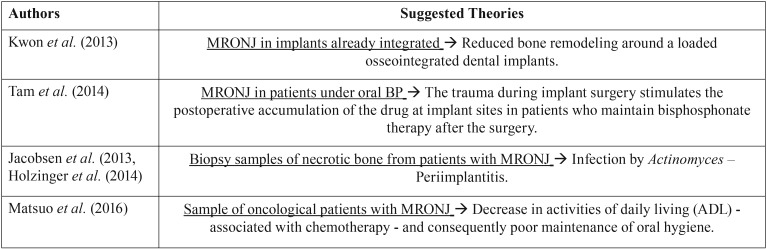
Suggested theories regarding implant placement, osteomodulating agents and MRONJ.

On the same subject Matsuo *et al.* with a sample of oncological patients, in treatment with intravenous bisphosphonates, identifies the decrease in activities of daily living (ADL) - associated with chemotherapy (the standard therapy for these patients) - and consequently poor maintenance of oral hygiene as one of the main causes for the development of osteonecrosis associated with dental implants (16).

The majority of osteonecrosis present in the studies reviewed are stage II or III, in which pain was the main presenting symptom, sometimes associated with signs of infection, such as swelling and purulent discharge (16,24,26). It is worthy of mention that not all studies classify Osteonecrosis in the stages proposed by the AAOMS, considering that stage I is usually asymptomatic it is possible to presume that some Osteonecrosis are underdiagnosed (16). Khoury *et al.* present very favorable results in their series of cases in which 15 patients in treatment with bisphosphonates and with severe bone atrophy were treated with bone grafts using mandibular bone blocks. The general results were similar to those of patients without bisphosphonate treatment. However, it should be mentioned that the authors selected the patients to be treated, assessing the risks of each patient, rejecting the regenerative treatment in high-risk patients. According to the authors, part of the success of their results is due to a correct anamnesis to evaluate the individual risk, a good surgical protocol, with primary closure of the wounds and with prophylactic and postoperative antibiotic treatment (32). None of the articles refers to the quality of prosthetic rehabilitation, except Holzinger *et al.* that cites this factor as one of the limitations of the study design (9). Only one article by Tam *et al.* mentions the professional’s experience as a possible risk factor for the development of Osteonecrosis associated with placement and implants (25). All the articles reviewed focus on the treatment with bisphosphonates, there being no studies at the time of writing this article that meet the inclusion / exclusion criteria on the role of other agents (anti RANKL, angiogenesis inhibitors) in osteonecrosis associated with implants / regenerations.

There is a consensus in the literature on the contraindication of implant placement in cancer patients treated with intravenous antiresorptive medication and a general favorable opinion about the treatment with dental implants in osteoporotic patients submitted to oral bisphosphonates, provided that an individual risk assessment is performed prior to surgery. The individual risk depends on the primary disease and its treatment, antiresorptive medication (substance, duration of application, and frequency of application), concomitant therapy and other risk diseases in implantology (diabetes etc). Other risk factors (not associated with antiresorptive medication) include smoking, advanced age, chronic physical inactivity, obesity, female patients and poor oral hygiene. It is essential to explain the potential risk of Osteonecrosis not only in patients under treatment with bisphosphonates who will undergo implant surgery but also in patients who already have osseointegrated implants and who must initiate antiresorptive therapy, due to the risk of developing a late “implant triggered” osteonecrosis.

In conclusion: i) The literature concerning this topic is scarce and consists mainly of clinical cases, series of cases and some retrospective studies. Randomized clinical trials are necessary to establish protocols for these patients. ii) The risk of developing an Osteonecrosis associated with regeneration and / or implant placement in patients with benign bone diseases is low, but it exists and should not be underestimated. Especially, and according to some authors, in the posterior areas of the jaw, if the duration of treatment with BP is greater than 3 years, and if the patient is under therapy with systemic corticosteroids. iii) An implant failure due to periimplantitis or osteonecrosis can present relatively similar clinical manifestations; however, the “en bloc sequestration” seems to be a pathognomonic feature of the MRONJ, thus allowing to correctly diagnose the cause of the failure. iv) A strict surgical protocol with primary closure of the wound, without tension, seems to reduce the risk of osteochimionecrosis and it is essential that patients attend periodic check-ups after medical-surgical treatment. v) There is not, at least until the writing of this article, literature that meets the inclusion criteria on patients under treatment with anti RANKL monoclonal agents and antiangiogenic drugs, so the recommendations are, for now, to proceed with these patients the same way as in patients with bisphosphonates, especially when considering that many of these patients, mainly the ones under Denosumab, were previously treated with bisphosphonates to control benign bone pathology. vi) The prescribing specialist must be aware of the risk and refer the patient to a dentist for evaluation before starting treatment, thus allowing the opportunity to offer the best rehabilitation treatment according to the patient osteomodulating therapy, and in case the patient already has osseointegrated implants, to warn and explain the risk of MROJ, ( Table 4).

**Table 4:**
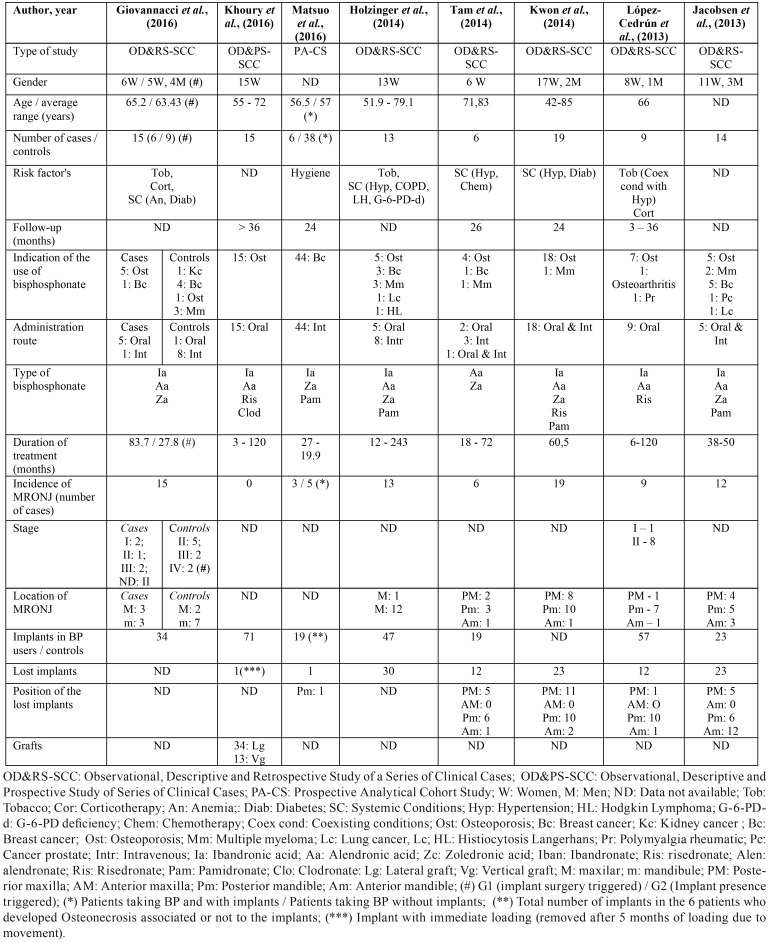
Summary of studies included in the review.
